# The opinion of French pulmonologists and palliative care physicians on non-invasive ventilation during palliative sedation at end of life: a nationwide survey

**DOI:** 10.1186/s12904-021-00755-w

**Published:** 2021-05-17

**Authors:** V. Guastella, G. Piwko, A. Greil, C. Lambert, A. Lautrette

**Affiliations:** 1 Palliative Care Unit, Montpied Hospital, University Hospital of Clermont-Ferrand, 54 rue Montalembert, BP69, 63003 Clermont-Ferrand, Cedex 1, France; 2 Pulmonology Unit, Montpied Hospital, University Hospital of Clermont-Ferrand, Clermont-Ferrand, France; 3 Biostatistics unit (DRCI), Montpied Hospital, University Hospital of Clermont-Ferrand, Clermont-Ferrand, France; 4 Medical Intensive Care, Montpied Hospital, University Hospital of Clermont-Ferrand, Clermont-Ferrand, France

**Keywords:** Palliative care, End of life, Non-invasive ventilation, Limitation of treatment

## Abstract

**Background:**

Deciding to withdraw non-invasive ventilation (NIV) at end-of-life (EOL) in patients with chronic respiratory failure is a challenge. The European Association for Palliative Care recommends not maintaining artificial therapies that could prolong life during palliative sedation (PS) at EOL. The aim of this survey was to assess palliative care physicians’ and pulmonologists’ opinion on withdrawing or maintaining NIV in patients with chronic respiratory failure during PS at EOL.

**Methods:**

From April to May 2019, we performed a prospective survey among pulmonologists (*n* = 1545) and palliative care physicians (*n* = 631) in France to determine the prevalence of opinion in favour of maintaining NIV and identify the factors associated with opinion in favour of withdrawing or maintaining NIV with multiple logistic regression.

**Results:**

A total of 457 participants were enrolled comprising 202 pulmonologists and 255 palliative care physicians. An opinion in favour of maintaining NIV was found in 88 (19.3 95%CI [15.7; 23.2]) physicians comprising 57 (28.2%) pulmonologists and 31 (12.2%) palliative care physicians (*p* < 0.001). The factors associated with an opinion in favour of maintaining NIV were spending time looking for advanced directives (AD) in the patient’s file (odds ratio (OR): 6.54, 95%CI [2.00; 21.32], *p* = 0.002*)* and personal ethics of physicians (OR: 17.97, 95%CI [9.52; 33.89], *p* < 0.001*).* The factor associated with an opinion in favour of withdrawing NIV was palliative care training (OR: 0.31, 95%CI [0.16; 0.60], *p* < 0.001)*.* The three main reasons in favour of maintaining NIV among the nine identified were emotional comfort for close relatives, reducing discomfort of dyspneoa and anticipation of suffocation.

**Conclusion:**

In France, around 20% of pulmonologists and palliative care physicians declared an opinion in favour of maintaining NIV during PS at EOL because of their personal ethics and spending time looking for AD, if any, in the patient’s file. Palliative care training can stimulate reflection help foster a change of opinion about practices, especially in the case of patients with NIV during PS at EOL.

**Supplementary Information:**

The online version contains supplementary material available at 10.1186/s12904-021-00755-w.

## Background

The aim of palliative care is retaining comfort until death without unreasonable obstinacy in patients with a severe critical illness in an end-of-life (EOL) setting. Whenever life-sustaining therapies are unable to improve a patient’s outcome, or paradoxically may prove more burdensome than beneficial, physicians must decide whether to withdraw or maintain therapies. The decision-making process during palliative sedation (PS) at EOL is standardized in the guidelines of the European Association for Palliative Care (EAPC), and favours not maintaining sustaining treatments when they prolong dying without improving the comfort of a patient with no hope of recovery [1–4]. Non-invasive ventilation (NIV) is a sustaining respiratory assistance in patients with chronic respiratory failure. Its use has greatly increased in the last few years [5] because it improves both prognosis and the quality of life and sleep [6]. At EOL, invasive therapies should be stopped because the main goal is then to enhance comfort. Dyspnoea is a frequent symptom in palliative situations with multifactorial aetiologies and a complex mechanism. In this context, therapies to alleviate dyspnoea show limited effectiveness. Finally, physicians are faced with refractory symptoms leading to asphyxia. NIV can alleviate symptoms [7, 8]. Some authors report that NIV improve the symptoms of acute respiratory failure in patients with contraindications for more invasive procedures such as orotracheal intubation [9, 10], It reduces the subjective sensation of dyspnoea by lessening respiratory muscular work [11]. Some others consider that maintaining NIV in a failing clinical condition may prolong dying unnecessarily [12]. Some patients describe NIV as an unpleasant therapy [13]. There is thus a choice between enhancing comfort by withdrawing or by maintaining NIV (as a way to relieve dyspnoea) [14].

In France, two laws, Leonetti 2005 [15] and Claeys Leonetti 2016 [14] regulate the procedures for withholding and withdrawing life-sustaining treatment at EOL. These laws proscribe unreasonable obstinacy. Some physicians may view the use of NIV at EOL as unreasonable obstinacy. A patient with chronic respiratory failure requiring NIV at EOL is managed by pulmonologists or palliative care physicians. The main difference between the two specialists is the timing in the care pathway. The palliative care physicians take care of patients at EOL. The pulmonologists take care of them from the onset of their respiratory disease and chronically. The follow-up by the pulmonologists is longer [13, 15]. At EOL, only one physician (pulmonologist or palliative care physician) takes care of the patient and communicates with the family.

The aim of this survey was to determine the prevalence of opinion in favour of maintaining NIV during PS at EOL among pulmonologists and palliative care physicians, who are the physicians taking care of patients with chronic respiratory failure. The secondary objective was to determine the factors associated with an opinion in favour of withdrawing or maintaining NIV.

## Methods

We conducted a prospective survey from April to May 2019 in all French pulmonologists (*n* = 1545) belonging to the French Pulmonology Society, and palliative care physicians (*n* = 631) belonging to the French Palliative Care Society. There were no exclusion criteria. This survey involving human participants, was performed in compliance with the Declaration of Helsinki and was approved by an ethics committee​: Comité de Protection des Personnes SUD EST VI (No. IRB 00008526, reference: 2020 / CE 77). An informed consent to participate in this survey was obtained from all the participants. The physicians were contacted by e-mail for enrollment and completed an attached questionnaire. The questionnaire was created by a multidisciplinary team made up of two pulmonologists and two palliative care physicians who were volunteered to participate in the survey including one professor and one physician at the end of their career, one physician in the middle of their career and one physician at the start of their career. The questionnaire was based on their own experience during brainstorming because no literature dealt with this subject.

Every participant completed an anonymous questionnaire (Supplementary File 1) including the physicians’ characteristics: gender, age, professional status, specific training in palliative care and/or NIV, at least one previous experience with the situation of EOL, unease in deciding to withdraw NIV, personal ethics in favour of maintaining NIV, and spending time looking for advanced directives (AD). Physicians were then asked whether their opinion was in favour of withdrawing or maintaining NIV during PS at EOL. The physicians with an opinion in favour of maintaining NIV were asked to state their reasons.

### Statistical analysis

The initial hypothesis was that 10–15% of physicians maintained NIV, determined with personal data, obtained from a survey, carried out by doctors (*n* = 20), on a given day, in our University Hospital.

Thus for a proportion of 15% (requiring the most subjects), 196 subjects would provide an accuracy of ±5%, and 307 subjects an accuracy of ±4%.

Statistical analysis was performed using Stata software (version 15; StataCorp, College Station, Texas, USA). All tests were two-sided, with a Type I error set at 0.05. All data were expressed as frequencies and associated percentages, except for the physicians’ age expressed as mean ± standard deviation. The number of physicians whose opinion was in favour of maintaining NIV was expressed as a rate with a 95% confidence interval (CI). Factors associated with maintaining NIV were studied using the chi-squared test or Fisher’s exact test for categorical variables, and Student’s *t*-test for quantitative ones (physicians’ age). A multivariate analysis was carried out using a logistic regression, considering the covariates according to univariate results and clinical relevance: training in palliative care, training in NIV, looking for AD, and personal ethics in support of maintaining NIV. The results were expressed as odds ratio (OR) and 95% CI. Comparisons according to the physicians’ speciality were made as described previously.

## Results

**Fig. 1:**
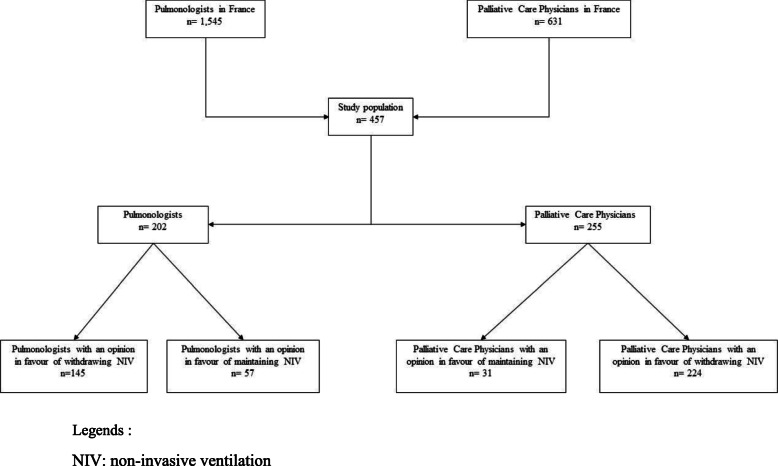
Flowchart of the survey. Legends: NIV: non-invasive ventilation

A total of 457 physicians were enrolled in the survey population, comprising 202 pulmonologists, representing 13.1% of all French pulmonologists, and 255 palliative care physicians, representing 40.4% of all French palliative care physicians (Fig. 1).

The overall prevalence of opinion in favour of maintaining NIV during PS at EOL was 19.3% in the survey population. The characteristics of physicians are shown in Table 1. The prevalence of opinion in favour of maintaining NIV during PS among the pulmonologists was significantly higher than among the palliative care physicians (28.2% vs. 12.2% respectively, *p* < 0.001).

**Table 1:** Pulmonologists’ and palliative care physicians’ characteristics

	Study population (***n*** = 457)	Pulmonologists (***n*** = 202)	Palliative care physicians (***n*** = 255)	***p***
Female gender	256 (56.0)	102 (50.5)	154 (60.4)	0.034
Age (years)	45.6 ± 12.1	44.3 ± 12.3	46.6 ± 11.8	0.040
Status of physicians				
Senior practitioner	437 (95.6)	188 (93.1)	249 (97.6)	0.018
Professor	20 (4.4)	14 (6.9)	6 (2.4)	
Training in palliative care	284 (62.1)	29 (14.4)	255 (100.0)	< 0.001
Training in NIV	161 (35.2)	138 (68.3)	23 (9.0)	< 0.001
Training in NIV and palliative care	44 (9.6)	21 (10.4)	23 (9.0)	0.62
Opinion in favour of maintaining NIV	88 (19.3)	57 (28.2)	31 (12.2)	< 0.001
Experience of NIV use at EOL with palliative sedation	277 (60.6)	133 (65.8)	144 (56.5)	0.042
Doctor uneasy in deciding to withdraw NIV	145/277 (52.3)	88/133 (66.2)	57/144 (39.6)	< 0.001
Personal ethics support maintaining NIV	71 (15.5)	39 (19.3)	32 (12.5)	0.048
Spending time looking for AD in the patient’s file	394 (86.2)	161 (79.7)	233 (91.4)	< 0.001

Data are presented as frequencies (associated percentages), or mean ± standard deviation

*EOL* end of life, *NIV* noninvasive ventilation, *AD* advanced directives

In univariate analysis (Table 2), the opinion in favour of maintaining NIV was associated with the pulmonology speciality, training NIV, unease in deciding to withdraw NIV after an experience in PS at EOL, personal ethics in support of maintaining NIV and the spending time looking for any AD in the patient’s file.

**Table 2:** Differences in characteristics between physicians with an opinion in favour of maintaining NIV and physicians with an opinion in favour of withdrawing NIV

	Withdrawing (***n*** = 369)	Maintaining (***n*** = 88)	***p***
Female gender	202 (54.7)	54 (61.4)	0.26
Age (years)	45.7 ± 12.1	45.0 ± 12.2	0.64
Status of physicians,			
Senior practitioner	351 (95.1)	86 (97.7)	0.39
Professor	18 (4.9)	2 (2.3)	
Speciality of physicians			
Pulmonologists	145 (39.3)	57 (64.8)	< 0.001
Palliative care physicians	224 (60.7)	31 (35.2)	
Training in palliative care	245 (66.4)	39 (44.3)	< 0.001
Training in NIV	122 (33.1)	39 (44.3)	0.047
Training in NIV and palliative care	37 (10.0)	7 (8.0)	0.55
Experience of NIV use at EOL with palliative sedation	217 (58.8)	60 (68.2)	0.11
Doctor uneasy in deciding to withdraw NIV	105/217 (48.4)	40/60 (66.7)	0.012
Personal ethics support maintaining NIV	24 (6.5)	47 (53.4)	< 0.001
Spending time looking for AD in the patient’s file	310 (84.0)	84 (95.4)	0.005

Data are presented as frequencies (associated percentages), or mean ± standard deviation. *EOL* end of life, *NIV* non-invasive ventilation, *AD* advanced directives

In the multivariable analysis, the factors associated with an opinion in favour of maintaining NIV were the spending time looking for any AD in the patient’s file and personal ethics physicians. The factor associated with an opinion in favour of withdrawing NIV was palliative care training. The training in NIV was not associated with an opinion in favour of withdrawing or maintaining NIV (Table 3).

**Table 3:** Multivariable analysis of the factors associated with an opinion in favour of maintaining non-invasive ventilation

	OR [95% CI]	***p***
Training in palliative care	0.31 [0.16; 0.60]	< 0.001
Training in NIV	0.83 [0.43; 1.61]	0.58
Spending time looking for any AD in the patient’s file	6.54 [2.00; 21.32]	0.002
Personal ethics support maintaining NIV	17.97 [9.52; 33.89]	< 0.001

*AD* advanced directives, *CI* confidence interval, *NIV* non-invasive ventilation, *OR* odds ratio

**Fig. 2:**
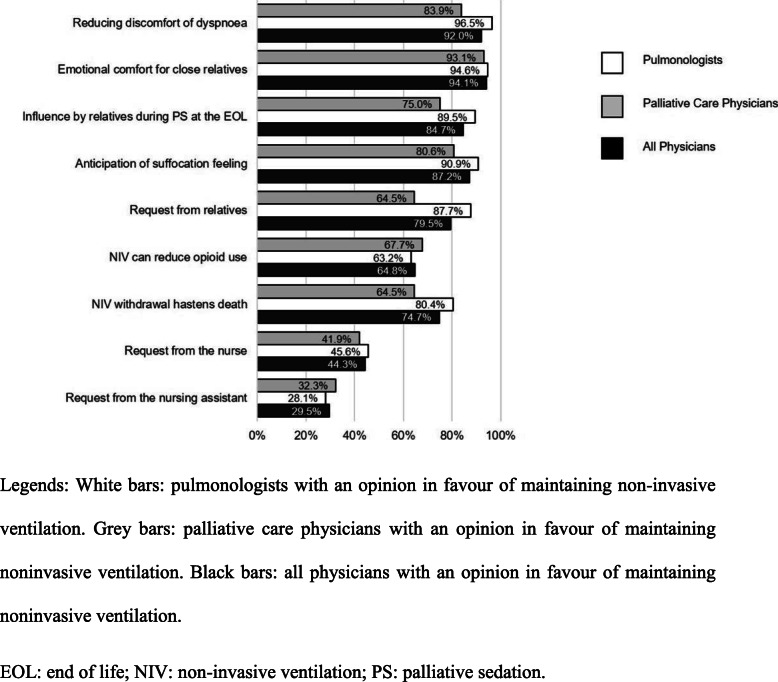
Reasons in favour of maintaining non-invasive ventilation among the 88 physicians in favour of maintaining it

Nine reasons in favour of maintaining NIV were identified among the 88 physicians with this opinion (Fig. 2). The three most prevalent reasons were emotional comfort for close relatives, reducing discomfort of dyspnoea, and anticipation of suffocation.

## Discussion

This survey performed in France shows that 20% of the practitioners still had favourable opinion of maintaining NIV during PS at EOL. Palliative care training was a factor associated with opinion in favour of withdrawing NIV in contrast to the research time for AD. Spending time looking for any AD in the patient’s file and personal ethics of physicians were associated with the opinion in favour of maintaining NIV during PS at EOL [16, 17]. Most often, patients’ preferences were unknown, and so decisions remained primarily based on medical judgment [18]. The consensus was that life-sustaining treatments should be maintained or withdrawn when they prolong dying without improving the comfort of a patient with no hope of recovery. Guidelines state that the same professional ethics apply to maintaining and to withdrawing life support [17, 18]. The decision-making process for treatment limitations is standardized in guidelines, although everyday practice is characterized by some variability related to social and religious factors, and the personal ethics of physicians, staff members, patients, and relatives [19, 20]. The French Claeys Leonetti law on the end of life (February 2, 2016) [14] entitles patients to ask for PS at EOL that induces decreased the awareness until death. This is put in place after a common decision in the case of a serious, incurable affection that is life-threatening in the short term.

Given the 20% rate of maintaining NIV found among doctors surveyed in this study, the important question that arises is why this rate is still so high when the law proscribes stubborn life support in this situation and how it can be lowered. We see that decisions and attitudes are dictated by the doctors’ personal ethics concerning the NIV without training in NIV and in palliative care. Doctors with training in palliative care are less likely to maintain NIV than those without. They are more aware of guidelines, and being trained they feel better able to make reasoned decisions in these types of medical situation. Pulmonologists are trained in NIV and know the benefits of this technical innovation and particularly the symptomatic comfort it brings to patients, and so are reluctant to withdraw it. Their behaviour is also different from that of palliative care physicians because they are so used to seeing their patients with NIV as a life support. NIV is an efficient chronic therapy for pulmonologists and they can minimize the discomfort related to NIV [21–23]. By contrast, the palliative care physicians prioritize comfort at EOL over respiratory therapy. The stage of patient care for pulmonologists and palliative care physicians is different, so the perception of therapy support with NIV by the two specialities may also be different. For the palliative care physicians, the NIV can be perceived as a cause of discomfort, whereas it remains a vital therapy for the pulmonologists. In our study, spending time looking for any AD in the patient’s file was identified as a factor associated with an opinion in favour of maintaining NIV. Further looking for AD into the patient’s file will enable to figure out the latter’s will. It is possible that the physicians with an opinion in favour of maintaining NIV want to validate their decision with the AD because they know that their opinion is in the minority.

There is also a significant influence of the family on doctors. Whereas caregivers undergo no such influence. Information about sustaining treatment and sedation practices should be given to every citizen because anyone can be faced with a similar situation. In this way, EOL could be better anticipated, rather than having to decide each time an acute situation arises.

### Limitations and strengths of the study

A binary question such as withdrawing or maintaining NIV during PS without any context except for the EOL is admittedly hard to answer. It requires ethical reflection, and it omits many important questions in regard to PS at EOL. The lack of context and the binary response design may thus skew the survey results.

Finally, we cannot claim that the survey sample is fully representative of all the French physicians caring for patients with NIV for chronic respiratory failure at EOL as this was an observational study.

A strength of the survey is that the professionals surveyed had very different training on the same practice during PS at EOL. This survey yielded up-to-date, detailed data on maintaining or withdrawing NIV, so opening numerous perspectives for research.

This survey is an opinion poll. It would be of interest to perform a prospective survey and study what really happens at the patients’ bedside. It is always difficult for a practitioner to take part in an opinion survey outside a real clinical situation.

## Conclusion

To sum up, our survey shows that in most cases, physicians had an opinion in favour of withdrawing NIV, but 20% still had a favourable opinion of maintaining it. We also found that palliative care training was a factor associated with an opinion in favour of withdrawing NIV, whereas spending time looking for any AD in the patient’s file and physicians’ personal ethics were associated with an opinion in favour of maintaining NIV. Scientific and technical skills are needed for proper care techniques, but so also are interpersonal, ethical, legal and reflexive skills.

## Supplementary Information

**Additional file 1.**

## Data Availability

The data that support the findings of this survey are available from the corresponding author upon reasonable request.

## Abbreviations

EOL: End of life
AD: Advanced directives
PS: Palliative sedation
NIV: Non-invasive ventilation
