# Supplementary material for: The opinion of French pulmonologists and palliative care physicians on non-invasive ventilation during palliative sedation at end of life: a nationwide survey
Source: BMC Palliat Care. 2021 May 17;20:68. doi: 10.1186/s12904-021-00755-w (PMC8130279; doi:10.1186/s12904-021-00755-w)
Supplement: Supplementary file 1 — Additional file 1. [file 12904_2021_755_MOESM1_ESM.docx]

**Questionnaire**

You are invited to fill a questionnaire for a national survey entitled **“What is the opinion of French pulmonologists and palliative care physicians on noninvasive ventilation during palliative sedation at end of life.”**

The study is approved by the Ethics committee (No. IRB 00008526, reference: 2020 / CE 77). It will take 5 minutes. The answers collected are anonymous.

- **What gender are you? female male**
- **How old are you? (years)**
- **What is your professional status?**

**Senior practitioner / Professor**

- **What is your speciality?**

**Pulmonologist / Palliative care physician**

- **Do you have an opinion in favour of maintaining NIV? yes no**
- **Do you have training in palliative care? yes no**
- **Do you have training in NIV? yes no**
- **Do you have experience of NIV use in patients at EOL**

**with palliative sedation? yes no**

- **If “Yes”, Do you feel uneasy deciding to withdraw NIV? yes no**
- **Do you have personal ethics that support**

**maintaining NIV? yes no**

- **Do you spend time looking for advanced directives**

**in the patient’s file? yes no**

**If you answered “Yes” to the previous question : “Do you have an opinion in favour of maintaining NIV “, could you tell us for what reasons you are in favour of maintaining NIV?**

- **Reducing discomfort of dyspnoea yes no**
- **Emotional comfort of close relatives yes no**
- **Influence by relatives during PS at EOL yes no**
- **Anticipation of suffocation feeling yes no**
- **Request from relatives yes no**
- **NIV can reduce opioid use yes no**
- **NIV withdrawal hastens death yes no**
- **Request from the nurse yes no**
- **Request from the nursing assistant yes no**

**Thank you for your participation**
